# Supplementary material for: Detailed morphological characterization and improvement of keratinocyte outgrowth from plucked human hair follicle
Source: PeerJ. 2025 Oct 31;13:e20214. doi: 10.7717/peerj.20214 (PMC12581916; doi:10.7717/peerj.20214)
Supplement: Supplemental Information 2 [file peerj-13-20214-s002.docx]

**Supplemental Table T1**

HF donors with different sex and age which were used in this study.

| **Donor** | **Sex** | **Age** |
| --- | --- | --- |
| K1 | male | 38 |
| K4 | male | 35 |
| K2 | female | 35 |
| K3 | female | 25 |
