# Supplementary material for: Detailed morphological characterization and improvement of keratinocyte outgrowth from plucked human hair follicle
Source: PeerJ. 2025 Oct 31;13:e20214. doi: 10.7717/peerj.20214 (PMC12581916; doi:10.7717/peerj.20214)
Supplement: Supplemental Information 3 [file peerj-13-20214-s003.docx]

**Supplemental Table T2**

List of different cell culture media and supplements used for HF cultivation in this study

| **Basic medium** | **Supplement** | **Supplier** | **Annotation** |
| --- | --- | --- | --- |
| MEF medium | DMEM | Thermo Fisher, #41965-039 | |
|  | + 10 % FBS | Thermo Fisher, #10500-064 | |
|  | + 100 µM NEAA | Thermo Fisher, #11140-050 | |
|  | + 2 nM GlutaMax | Thermo Fisher, #35050-038 | |
|  | + 1 x Anti-Anti | Thermo Fisher, #15240-062 | |
|  | + 10 ng/ml FGF2 | Cell Guidance, #GFH146-50 | Freshly added |
|  | + 10 µM Y-27632 | Selleck Chem, #S1049 |  |
| EpiLife | Thermo Fisher, #M-EPI-500-CA | | |
|  | + 1 x HKGS | Thermo Fisher, #S-001-5 | |
|  | + 10 µM Y-27632 | Selleck Chem #S1049 | Freshly added |
