# Supplementary material for: Detailed morphological characterization and improvement of keratinocyte outgrowth from plucked human hair follicle
Source: PeerJ. 2025 Oct 31;13:e20214. doi: 10.7717/peerj.20214 (PMC12581916; doi:10.7717/peerj.20214)
Supplement: Supplemental Information 4 [file peerj-13-20214-s004.docx]

**Supplemental Table T3**

List of cell culture supplements used in this study

| **Supplement** | **Concentration** | **Supplier** | **Annotation** |
| --- | --- | --- | --- |
| A83-01 | 10 µg | Merck Milipore, #616454 | Freshly added |
| DAPT | 10 µg | STEMCELL Technologies, #72082 |  |
| IGF1 | 20 ng/ml | PeproTech, #100-11 |  |
| EGF | 20 ng/ml | PeproTech, #100-47 |  |
| SB431542 | 10 µg | Merck Milipore, #301836-41-9 |  |
