# Supplementary material for: Detailed morphological characterization and improvement of keratinocyte outgrowth from plucked human hair follicle
Source: PeerJ. 2025 Oct 31;13:e20214. doi: 10.7717/peerj.20214 (PMC12581916; doi:10.7717/peerj.20214)
Supplement: Supplemental Information 5 [file peerj-13-20214-s005.docx]

**Supplemental Table T4**

List of blocking solution and antibodies used for immunostaining in this study

| **Blocking solution** | | **Supplier** |
| --- | --- | --- |
| DPBS^-/-^ | Thermo Fisher, #14040133 | |
|  | + 10 % Normal donkey serum | Merck Milipore, #566460 |
|  | + 5 % Bovine serum albumin | VWR, #9048-46-8 |
|  | + 4 % skimmed milk solution | TSI GmbH, #Sucofin |
|  | + 0,1 % Triton-X | Carl Roth, #3051.1 |
|  | | |
| **Primary antibodies** | | **Dilution, Supplier** |
| Cytokeratin 5 | | 1:100, Biolegend, #905501 |
| Cytokeratin 6 | | 1:50, Santa Cruz, #sc-514520 |
| Cytokeratin 6/75 | | 1:50, Santa Cruz, #sc-166074 |
| Cytokeratin 10 | | 1:50, Santa Cruz, #sc-23877 |
| Cytokeratin 14 | | 1:100, Thermo Fisher, #MA5-11599 |
| Cytokeratin 15 | | 1:50, Santa Cruz, #sc-47697 |
|  | | |
| **Secondary antibodies** | | **Dilution, Supplier** |
| Donkey anti-rb IgG Alexa Fluor488 | | 1:1000, Thermo Fisher, #A32790 |
| donkey anti-ms Alexa Fluor546 | | 1:1000, Thermo Fisher, #A10036 |
| donkey anti-gt Alexa Fluor647 | | 1:1000, Abcam, #ab150131 |
